# Frequency and reasons for missed appointments of outpatient mental health care users in the uMgungundlovu District

**DOI:** 10.4102/curationis.v41i1.1835

**Published:** 2018-07-31

**Authors:** Lucelle Ramlucken, Maureen N. Sibiya

**Affiliations:** 1 KwaZulu-Natal Department of Health, South Africa; 2 Department of Nursing, Durban University of Technology, South Africa

## Abstract

**Background:**

Over the years, there has been a rapid growth in the use of mobile technology which has been proven to increase treatment adherence. Short message services may improve service delivery through appointment reminders and improve communication between health care workers and patients. Missed appointments are becoming common amongst mental health care users, and this has a significant economic burden on mental health symptoms.

**Objectives:**

The aim of the study was to determine the frequency and reasons for missed appointments of outpatient mental health care users for their follow-up care in the uMgungundlovu District.

**Method:**

This study used a quantitative survey. A non-probability convenient sampling method was used to select 182 participants at the psychiatric clinics.

**Results:**

Of the 182 participants, results of the study indicated that *n* = 84 (46.2%) respondents had missed their appointment at some stage. Of the *n* = 84 (46.2%) respondents who had missed appointments, *n* = 28 (33.3%) had missed their appointment once, and *n* = 45 (53.6%) had missed their appointment 2–3 times. Most common reasons for missed appointments included mental health care users forgetting (*n* = 58; 69%), work commitments (*n* = 14; 16.7%), no transportation (*n* = 4; 4.8%) and financial constraints (*n* = 5; 6%).

**Conclusion:**

The main reasons for missed appointments that were identified included forgetfulness, work commitments, lack of transportation and financial constraints. A significant number of participants (53.6%) had missed their appointments 2–3 times.

## Introduction

According to the World Health Organization (WHO) (2004:2), mental health is a state of well-being in which the individual realises his or her own abilities, can cope with the normal stress of life, can work productively and fruitfully, and is able to contribute to his or her own community. To ensure improved mental health for mental health care users, attending treatment sessions is very important.

Defife et al. (2010:415) stated that treatment attendance is often poor with psychotherapy appointment no-shows showing that 13% of scheduled appointments were non-attended, with most of the patients who missed appointments missing one appointment. Frequently missed appointments by mental health care users (MHCUs) lead to clinical and administrative challenges for staff and the MHCU (Defife et al. 2010:413–417). Cheng et al. (2012:439) concur that missed appointments interrupt the treatment programme and this may increase the cost to mental health systems. A study conducted by Branson, Clemmey and Mukherjee (2013:298) in the USA found that missed appointments were common barriers to treatment. This becomes a burden for community mental health centres owing to long waiting lists, lost revenue and the amount of time that staff spends on outreach.

Alhamad (2013:258–259) states that non-attendance is gaining attention, and missed appointments seem to have serious consequences, both economically and clinically. Often little is known about why appointments are missed, adding to their patient load. The following are the most frequently mentioned reasons for missed appointments: work commitments, travel, difficulty making an appointment, transportation and financial difficulties, struggling to book an appointment, child care responsibilities and forgetting the appointment (Alhamad 2013:264–266). This is also confirmed by Neal et al. (2005:47) who stated that individuals who missed an appointment disclosed that they had forgotten the appointment, had family commitments, had appointments at an inconvenient time, had made mistakes and had misunderstandings. A pilot study by Lim et al. (1995:403) found that the largest proportion claimed to have work commitments, have felt physically unwell, had forgotten their appointments or had lost their appointment cards. The majority of the participants in the study suffered from schizophrenia (Lim et al. 1995:404). Defife et al. (2010:415) found similar reasons for missed appointments.

Various strategies have been suggested as interventions to improve attendance, with Seko et al. (2014:591–602) in Canada recommending text message reminders for appointments. According to Jack and Mars (2014:2), there has been a rapid growth of mobile phone penetration in Africa. Two-thirds of the population have an active subscriber identity module (SIM) card. South Africa ranks third in Africa when it comes to mobile phone penetration. In South Africa, there has been a rapid growth in cellular devices, and this has created an opportunity for them to be used in health interventions. Norris, Swartz and Tomlinson (2013:379–388) indicate that mobile phones are becoming more accessible to South Africans because of the reduction in prices. This makes South Africa the perfect platform for mHealth projects. There is little data regarding mobile technology in the mental health field in South Africa, and Norris et al. (2013:380) believe that mobile technology is an important option to address the mental health treatment gap in South Africa.

## Research aim

The aim of this research was to determine the frequency and reasons for missed appointments of outpatient MHCUs in the uMgungundlovu District.

### Research method and design

### Study design

This study employed a quantitative, descriptive survey.

### Setting

The study took place at four outpatient psychiatric clinics in the uMgungundlovu District in KwaZulu-Natal. The four psychiatric clinics are based in Pietermaritzburg. One psychiatric clinic is based in a specialised hospital in the uMgungundlovu District.

### Study population and sampling strategy

The numbers of MHCUs that collect treatment from the four clinics total on average about 725 per month. Participants who were 18 years and above, participants who owned a cell phone and participants who were utilising the mental health facility and are mentally healthy were included in the study. Access to the participants was limited because participants were selected as they presented themselves for follow-up appointments and as the researcher was unable to locate the entire population. In consultation with a statistician, estimating the sample, a quarter sample would provide 182 MHCUs that visited in a week. This equates to sampling every patient that came in over a week. This seemed reasonable as patients from one week to the next should not be that different. Each clinic has a specific day to attend to MHCUs, and they are booked for that day. The researcher presented at the clinic on that specific day. Data were therefore collected within a week. The sample size of 182 was considered a reasonable size for analysis. To select the MHCUs, the convenient method of sampling was used.

### Pre-testing of the data collection tool

A pre-test survey was conducted by the researcher on eight participants who met the inclusion criteria. A survey was used to generate data, which were adapted from Dr Caron Jack’s study. Permission was obtained from Dr Caron Jack. The survey was conducted through utilising a structured questionnaire.

### Data analysis

The analysis was carried out using SPSS, version 23. Descriptive statistics in the form of tables and graphs were used to describe the data graphically. In order to test for significant trends in the data, inferential statistics were applied. Statistical tests were conducted to establish the reasons and frequency for missed appointments. These included Pearson’s chi-squared test, *t*-tests, ANOVA and chi-square goodness-of-fit-tests. Where the conditions were not met for the application of these tests, non-parametric equivalent tests or exact tests were used. Throughout, a *p*-value of 0.05 was used to indicate significance.

### Ethical considerations

Ethical approval was obtained from the Institutional Research Ethics Committee of Durban University of Technology (REC107/16) and the Health Research & Knowledge Management (HRKM Ref: 089/17). Permission was sought from and granted by the District Manager of uMgungundlovu Health District, KwaZulu-Natal Department of Health and the Chief Executive Officer of the selected hospital. The three ethical principles guided the researcher during the research process, which are (1) respect for persons, (2) justice and (3) beneficence.

## Results

### Key findings

#### Demographic data

The response rate from participants was 100%. The majority of the respondents were females (*n* = 94; 51.6%), and the ages ranged between 18 and 83 years, average age was 45.94. The majority of respondents lived in other areas (*n* = 78; 42.9), but most of the respondents could communicate in English (*n* = 174; 95.6%).

Of those who knew their diagnosis (*n* = 146), *n* = 46 (31.5%) indicated that they had been diagnosed with psychotic disorder; *n* = 40 (27.4%) had anxiety disorder; *n* = 32 (21.9%) suffered with bipolar disorder; *n* = 61 (41.8%) suffered from depression; *n* = 10 (6.8%) were diagnosed with cognitive disorder; *n* = 6 (4.1%) had an eating disorder and *n* = 18 (12.3%) were diagnosed with epilepsy. Only one respondent suffered from somatic symptoms and related disorders, as well as adjustment disorder.

#### Reasons and frequency of missed appointments

The results of the study as outlined in Table 1 revealed that out of 182 respondents, *n* = 84 (46.2%) had missed appointments (*p* < 0.0005). Of the *n* = 84 (46.2%) respondents who had missed appointments, *n* = 28 (33.3%) had missed their appointment once, and *n* = 45 (53.6%) had missed their appointment 2–3 times within a 12-month period. Of the *n* = 84 (46.2%) respondents who had missed appointments *n* = 58 (69%) indicated that they had forgotten (*p* < 0.0005); *n* = 5 (6%) of respondents had no money (*p* < 0.0005); *n* = 4 (16.7%) respondents had no transport (*p* < 0.0005); and *n* = 14 (16.7%) of respondents were working (*p* < 0.0005). Other reasons specified for missed appointments included having to fetch a grandchild (*n* = 1; 1.2%; *p* < 0.0005); respondent was on holiday (*n* = 1; 1.2%; *p* < 0.0005); and respondent was sick (*n* = 1; 1.2%; *p* < 0.0005).

**TABLE 1:** Frequency of missed appointments.

Variables	Frequency (*n*)	Percentage (%)
Once	28	33.3
2–3 times	45	53.6
4–6 times	7	8.3
> 6 times	4	4.8

**Total**	**84**	**100**

## Discussion of key findings

### Demographic data

The findings of the study revealed that age may not be a barrier to technology. The majority of respondents lived in other outlying areas (*n* = 78; 42.9%); hence, respondents travelled a distance to receive follow-up care at the clinic. As a result, this could imply financial implications for a missed appointment. A significant proportion of respondents could communicate in English (*n* = 174; 95.6%). This does not necessarily indicate that respondents could read English. However, the questionnaires were completed by the respondents themselves, indicating that they could read English. Only a small proportion of *n* = 8 (4.4%) could not communicate in English. These respondents completed the questionnaire in isiZulu. Little attention has been devoted to the impact of language on follow-up care.

Results of this study indicate that there were 146 respondents who knew their diagnosis, with a significant *n* = 46 (31.5%) diagnosed with a psychotic disorder; *n* = 61 (41.8%) with depression; *n* = 40 (27.4%) suffering from anxiety; and a significant *n* = 32 (21.9%) diagnosed with bipolar disorder. Cognitive impairment is common.

### Missed appointments

Mental health care users in this study may not perceive the seriousness of their diagnosis as a serious threat as *n* = 58 (69%) indicated that they had forgotten (*p* < 0.0005). Respondents who were working accounted for *n* = 14 (16.7%) of the sample (*p* < 0.0005). Findings from Alhamad (2013), Lim et al. (1995) and Neal et al. (2005) indicated forgetfulness and work as contributing factors to missed appointments. Although Firth et al. (2016:449) mention that unemployment reduces access to technology, results of this study indicate otherwise. Of the *n* = 181 participants (99.5%) who owned and used mobile phones, a significant *n* = 140 (77.3%; *p* < 0.0005) indicated that they run their cell phones on a ‘pay as you go’ basis, and *n* = 35 (19.3%) had a mobile phone contract. A significant number of participants (*n* = 83; 45.9%; *p* < 0.0005) had been out of airtime for more than a week. Participants who were working accounted for *n* = 14 (16.7%; *p* < 0.0005) of the sample, which could possibly account for *n* = 35 participants (19.3%) who had mobile phone contracts.

### Financial constraints

Respondents who had no money to attend follow-up care accounted for *n* = 5 (6%) of the sample (*p* < 0.0005), and *n* = 4 (16.7%) had no transport (*p* < 0.0005). Participants in Alhamad’s (2013:264) study indicated that transportation was a challenge, especially for unemployed females, with no formal education. Participants often have to wait for a chaperon in order to attend clinic appointments, as it is not socially acceptable for females to use taxis.

### Limitations of the study

There were several limitations to the study. Firstly, results were obtained from MHCUs attending psychiatric community clinics and a tertiary outpatient department where medication is supplied from one pharmacy department. There are numerous other clinics that have their medication dispensed to them by district hospitals. Frequency and reasons for missed appointments may prove to be different at those clinics, as they are situated in different areas, with varied resources. In relation to this, another limitation was that the sample size of 182 participants was small. A vast number of MHCUs attend outlying clinics for follow-up care, but these are not designated psychiatric clinics. Mental health care users attend general clinics and are not accounted for as MHCUs.

There may be some degree of respondent bias owing to familiarity with the researcher. The researcher can assume that there could have been some pressure to participate. Respondents could have felt intimidated not to participate and/or could have completed the questionnaire incorrectly.

## Recommendations

### Recommendations to the Department of Health

As health care providers, the main aim is to ensure that patients receive the best quality care, prevention of relapse and inpatient care. Encouraging patients to attend follow-up appointments will prompt MHCUs to be treatment compliant, allow for early detection of extra-pyramidal side effects caused by the use of psychotropic medication and a reduction in inpatient care.

Based on the results of this study, this accentuates the realisation that a policy for development and implementation of a short message service (SMS) system be considered, in order to remind MHCUs of appointments. Not only will this have positive health outcomes for MHCUs, but it will also inadvertently contribute to a decrease in economic burden, as consequences of poor health can be substantial for the economy.

It is recommended that an electronic patient database be initiated at each clinic site in order to keep health records of all patients, and for this database to be regularly updated with patients’ personal details. The researcher suggests that mental health care practitioners be trained and have access to digital technology in order to communicate effectively with MHCUs. Mental health campaigns and programmes need to be encouraged, with the main aim of reducing stigma.

### Nursing education

Mental health nurses should be encouraged to be able to communicate in the language of choice in the clinics they are working at. A short language course is recommended. This will aid in bridging the gap of a language barrier.

### Further research

The researcher postulates that this topic warrants further research. This study is limited in its generalisability owing to the small sample size. Therefore, it is suggested that a larger feasibility study is conducted. This should include district hospitals and outlying clinics to account for the large number of MHCUs who do not attend psychiatric hospitals and clinics.

## Conclusion

The study demonstrated that there were varied reasons as to why MHCUs missed follow-up appointments at the outpatient departments.

The results from this study are consistent with various studies that showed missed appointments as being common amongst psychiatric patients. SMS reminders for follow-up care may be an effective method in improving appointment attendance and treatment adherence. Although a significant proportion of respondents had indicated that they had not missed appointments or forgot to collect treatment, the relapse and admission rate remains high, reducing quality of life and incurring high economic cost for inpatient care.
